# Examining the application of behaviour change theories in the context of infectious disease outbreaks and emergency response: a review of reviews

**DOI:** 10.1186/s12889-020-09519-2

**Published:** 2020-10-01

**Authors:** Dale Weston, Athena Ip, Richard Amlôt

**Affiliations:** 1 Behavioural Science Team, Emergency Response Department Science & Technology, Public Health England, Porton Down, Salisbury, UK; 2 Primary Care and Population Sciences Division, University of Southampton, Southampton, UK

**Keywords:** Infectious disease, Emergency response, Human behaviour, Literature review, Protective behaviour

## Abstract

**Background:**

Behavioural science can play a critical role in combatting the effects of an infectious disease outbreak or public health emergency, such as the COVID-19 pandemic. The current paper presents a synthesis of review literature discussing the application of behaviour change theories within an infectious disease and emergency response context, with a view to informing infectious disease modelling, research and public health practice.

**Methods:**

A scoping review procedure was adopted for the searches. Searches were run on PubMed, PsychInfo and Medline with search terms covering four major categories: behaviour, emergency response (e.g., infectious disease, preparedness, mass emergency), theoretical models, and reviews. Three further top-up reviews was also conducted using Google Scholar. Papers were included if they presented a review of theoretical models as applied to understanding preventative health behaviours in the context of emergency preparedness and response, and/or infectious disease outbreaks.

**Results:**

Thirteen papers were included in the final synthesis. Across the reviews, several theories of behaviour change were identified as more commonly cited within this context, specifically, Health Belief Model, Theory of Planned Behaviour, and Protection Motivation Theory, with support (although not universal) for their effectiveness in this context. Furthermore, the application of these theories in previous primary research within this context was found to be patchy, and so further work is required to systematically incorporate and test behaviour change models within public health emergency research and interventions.

**Conclusion:**

Overall, this review identifies a range of more commonly applied theories with broad support for their use within an infectious disease and emergency response context. The Discussion section details several key recommendations to help researchers, practitioners, and infectious disease modellers to incorporate these theories into their work. Specifically, researchers and practitioners should base future research and practice on a systematic application of theories, beginning with those reported herein. Furthermore, infectious disease modellers should consult the theories reported herein to ensure that the full range of relevant constructs (cognitive, emotional and social) are incorporated into their models. In all cases, consultation with behavioural scientists throughout these processes is strongly recommended to ensure the appropriate application of theory.

## Background

The United Kingdom (UK) National Risk Register details a broad range of threats to the public health and security of the UK incorporating infectious disease outbreaks (e.g., pandemics and emerging diseases), malicious attacks (e.g., terrorist incidents), and natural phenomena (e.g., extreme weather, earthquakes) [1]. The risk of infectious disease outbreaks is so substantial that the UK National Risk Register ranks a pandemic outbreak as the number one high consequence civil emergency facing the UK (based on likelihood and probable impact [1]. The coronavirus disease (COVID-19) pandemic which, at the time of writing has led to 19,440,423 confirmed cases and 722,706 deaths, presents a stark reminder of this public health threat [2].

Outbreaks of infectious disease, particularly those for which little or no pre-existing immunity exists – such as the COVID-19 pandemic – represent a significant risk to public health. For example: the 2013–2016 Ebola outbreak in West Africa led to over 28,600 cases with 11,325 deaths [3], while the ongoing outbreak in the Democratic Republic of Congo has led to over 2200 deaths thus far [4]; since the identification of Middle East respiratory syndrome coronavirus (MERS-CoV) in 2012, 851 associated deaths have been reported with cases across 27 countries [5], and; although less severe than expected [6], the H1N1 pandemic was estimated as responsible for between 151,700–575,400 deaths worldwide during the first 12 months [7, 8].

Even when controlling for confounding factors (e.g., improvements in surveillance, communication infrastructure, etc.), the number of infectious disease outbreaks has substantially increased since 1980 to 2013 [9]. Similarly, deaths from terrorism have substantially increased from less than 200 in 1970 to over 26,000 in 2017 (peaking with over 44,000 in 2014 [10]), and despite a decline in the number of individuals affected by natural disasters between 1994 and 2014, the average death rate has increased over the same time period [11]. Given these trends, it is therefore critical to ensure that emergency preparedness, response and resilience is optimised to mitigate the occurrence and/or impact of these events.

Behavioural science represents one such broad method of mitigation. The importance of encouraging adaptive and protective behaviour change in response to public health emergencies is emphasised by the World Health Organisation (WHO), who provide risk communications guidelines designed to encourage individuals, families, and communities to act to protect themselves [12]. This is echoed in the context of COVID-19, with Michie and colleagues stating that: “human behaviour will determine how quickly covid-19 spreads and the mortality. Therefore behavioural science must be at the heart of the public health response” [13].

Research in the behavioural sciences has focused on identifying barriers and facilitators to maximising public compliance with recommended emergency response and infection prevention behaviours. For example, decontamination behaviour (e.g. [14]), medication adherence (e.g., [15]), hand washing (e.g., [16]), social distancing/ avoidance behaviour (e.g., [16, 17]), and vaccination (e.g., [18, 19]), to name but a few. Furthermore, in the context of infectious disease emergencies, mathematical models are used to both: a) understand and map out the spread and control of disease (incorporating human-to-human transmission) and, b) calculate the potential effectiveness of interventions (including behavioural interventions) to reduce the spread of the disease [20]. Considered together, the importance of human behaviour for emergency response – both in terms of developing interventions and its relevance for modelling the potential efficacy of said interventions – is clear.

However, there is still work to be done to optimise the incorporation of behavioural constructs in public health research, intervention design, and modelling. For example, despite Medical Research Council Guidelines recommending interventions be based on appropriate behaviour change theory [21] (see also [22]), reference to theory is often absent in such interventions [22]. Indeed, Michie and colleagues, pioneers in the field of identifying and integrating behaviour change theory and techniques in the context of health promotion, note that much intervention design is based on the principle of “It Seemed Like A Good Idea At The Time”, rather than a systematic consideration and assessment of the most appropriate routes to behaviour change ([23], p. 14).

Similarly, a limitation of traditional mathematical models is that they often do not allow for heterogeneous behavioural responses within a population [24]. This assumption that human behaviour is homogenous can impact on the validity of these models. For instance, including a modest degree of fear-related flight behaviour (i.e., 10% of individuals in a model respond to fear of infection with flight) into a model in which fear of infection otherwise leads to hiding, caused projected disease incidence to rise to ~ 65%, up from ~ 30% in a model in which fear of infection led all individuals to hide [25]. Although some recent infectious disease models do incorporate social and cognitive predictors of the kinds of self-protective health behaviours that are associated with infectious disease control and emergency response (e.g., vaccination uptake, social distancing etc.), they are more commonly informed by literature from behavioural economics than psychology [26].

That is not to say that the integration of theory is a silver bullet for the success of mitigation strategies and modelling. For example, there is mixed evidence concerning the efficacy of theory-based interventions (see [22], p21 for a summary), inconsistency that may be based on the relevance of the chosen theory for the behaviour in question [22]. To illustrate this point, according to Michie and colleagues, there are a total of 83 behaviour change theories across the behavioural and social sciences [22]. Over the past three decades, multiple review papers and books have attempted to identify trends in theory use including those most frequently applied (e.g., [22, 27–30]). Despite some commonalities in underlying psychological processes (Michie and colleagues cede that many of the 1659 constructs identified within their book were different labels for overlapping constructs, [22]), this proliferation of competing theories and recommendations could indeed make it difficult for researchers, intervention designers, and modellers to decide which theories to use and in what context.

This unfortunately leads to a catch-22 situation: we wish to encourage non-specialists to use appropriate psychological theories and approaches within their own disciplines, yet we fail to recognise the complex and confusing landscape of psychological theory. Michie and colleagues have made great strides to simplify the process by which psychological theories are used to inform behavioural interventions [23]. However, there are still a large number of behaviour change theories that were designed with specific applications in mind: for example, the Behavioural-Ecological Model of Adolescent AIDS Prevention [31], the Integrated Theory of Drinking Behaviour [32], or the Social Ecological Model of Walking [33] Public health researchers, infectious disease modellers, and practitioners may therefore be understandably perplexed as to how best to model and examine or influence behaviour in the specific context of infectious disease outbreaks or emergency response.

This current paper therefore seeks to present a synthesis of the behaviour change theories that are most commonly applied within an emergency response or infectious disease outbreak context. That is, focused specifically on using behaviour change theories to understand and influence individuals’ engagement with protective health behaviours that are recommended during infectious disease outbreaks and public health emergencies. To identify these commonly applied theories, we conducted a scoping review of the existing literature, but with a particular focus on identifying reviews using behaviour change theory in an infectious disease or emergency response context. This approach is recognised as a method of distilling a substantial literature into a manageable summary of evidence for decision makers ([34], see also [35]). Although using this ‘review of reviews’ approach focused on secondary sources, which may have led to some relevant information being missed, it enabled us to reduce the quantity of papers identified in a large and highly diverse literature to a manageable level while still achieving a broad overview of the state of the art within the field. By dovetailing with Weston and colleagues’ recent review of the application of human behaviour within infectious disease models [26], the outcomes from this current review will enable us to make useful recommendations as to how psychological constructs, theory, and research can be used by public health practitioners, researchers modellers, to improve our understanding of human behaviour within the contexts of infectious disease outbreaks and emergency response.

## Methods

A scoping approach was adopted for our search. Scoping reviews are recommended as a mechanism by which a given literature might be summarised for policy makers or practitioners [36]. As the aim of this review was to summarise and synthesise the psychological literature on behaviour change to inform recommendations for public health researchers and modellers, the adoption of a scoping review framework was a logical and appropriate choice.

### Original search strategy

The literature search was conducted using PubMed, PsychInfo and Medline databases on the 6th January 2016. The databases were selected based on their coverage of discipline and context specific literature. Each database was searched individually to ensure that all Medical Subject Heading (MeSH) terms were used effectively. The search terms covered four major categories: behaviour, emergency response (e.g., infectious disease, preparedness, mass emergency), theoretical models, and reviews. Supplementary Information 1 provides the full list of search terms used for each database. Within the theoretical model category, we a priori selected several existing behaviour change models that were either: a) frequently cited within the literature, or b) adjudged to be of particular relevance within the context of infectious disease and emergency preparedness based on the authors combined expertise in these areas. In addition, generic phrases and subject headings for theoretical modelling were included within each search strategy to ensure that papers that do not cite the most common behaviour change models would still be captured within our search. Lastly, papers identified through other, non-systematic methods (e.g., some clearly relevant citations in papers, keyword Google Scholar searches) were also included to try to identify articles that were not indexed within these databases.

### Top-up search strategy

As the initial search was run in 2016, a condensed follow up search strategy was devised to identify seminal works in the field published since this date. Given time and resource constraints in conducting this search, the strategy was designed to identify literature that closely corresponded to the output from the original selection process. For example, the strategy was simplified based on the broad search categories used in the original search, and as literature concerning Human Immunodeficiency Virus (HIV)/ Sexually Transmitted Infections (STIs) was excluded from the original data extraction (see Inclusion/ Exclusion criteria section), the decision was taken to exclude these papers at the search strategy stage here. On 24/10/19 the following search was conducted on Google Scholar, sorted by relevance, with a custom date range of 2016–2019:

“review* AND behavio* AND theor*, OR models AND infectious disease*, OR emergenc* -HIV, -STD, -STI”Given time and resource constraints, only the first 20 pages of Google Scholar were screened, first for title, then for abstract, and finally full-text screening was conducted on any remaining papers.

### Optimised top-up search strategy

Due to limitations concerning the use of wildcard operators (*) on Google Scholar in the initial top-up search, and the potential for COVID-19 related review papers to have been published in the intervening period, a further optimised top-up search strategy was developed. This strategy consisted of the following two searches (specified for emergency response and infectious diseases respectively), conducted on Google Scholar on 16th – 17th May 2020:

review emergency theory behavior OR behaviour.

review disease theory behavior OR behaviour.

As for the previous top-up search, the first 20 pages of Google Scholar were screened for each search (for a total of 400 results).

### Study selection

For the original search, duplicates were removed electronically, and all remaining papers were subjected to title and abstract screening by one author (AI) using the inclusion/ exclusion criteria. All papers retained for full text assessment were screened independently by two researchers (AI and DW) to increase the reliability of the selection process. Any inconsistencies between the researchers were resolved through a joint discussion.

For the top-up search, individual title, abstract, and full-text screening stages were conducted by the first author (DW). As for the original search, all papers retained for full text assessment were screened independently by two researchers (AI and DW) with any inconsistencies between the researchers resolved through a joint discussion. The inclusion/ exclusion criteria employed in the top-up search were the same as those used for the original search.

For the optimised top-up search, individual title, abstract, and full-text screening stages were conducted by the first author (DW). Due to time constraints imposed by the COVID-19 pandemic, full text screening for this search was conducted by the first author alone. As for the initial top-up search, the same inclusion/ exclusion criteria were employed as used in the original review.

### Inclusion/exclusion criteria

The following inclusion/ exclusion criteria were used:

(i) Type of article: Reviews (systematic, scoping and narrative) and meta-analysis.
(ii) Theoretical model/theory: The papers needed to present or apply a model or theory of behaviour change. Leniency in this criterion was initially applied in so far as papers which clearly applied constructs that were adapted from theories/models, were also retained, but this was subsequently restricted to focus specifically on the presentation of behaviour change models/ theories (see Original Study Selection section below).
(iii) Context: The papers needed to present or apply the theory/model to explain human behaviour in the context of emergency or infectious disease outbreaks. As per [26] any reviews focusing on diseases that are not transferred from human-to-human (e.g., vector borne) were excluded.
(iv) Target behaviour: Preventive health behaviours during an emergency or outbreak (e.g. social distancing, vaccination and reducing social ties) were included in the review.
(v) Other: There were no restrictions on the date reviews were published or the population in question. Reviews were included if they were written in the English language and involved human behaviour.

For simplicity, papers exploring STI-related health behaviours (total: 53) were pragmatically excluded wholesale following the initial screening of the original search as the majority were deemed either: irrelevant according to the above criteria (30), of unclear relevance to the researcher (AI) (6), or inaccessible to the researcher (AI) (15). For consistency, papers exploring STI-related health behaviours have also been excluded during subsequent screening of the top-up searches.

### Data extraction

Based on data extracted as part of previous review work in this area (e.g., [27, 28]), the following information was extracted from the included papers in both the original and top-up stages:

(i) Title
(ii) Author
(iii) Number of studies included in the review
(iv) Target behaviour(s)
(v) Theories employed
(vi) Key outcomes/ conclusions regarding the utility of behaviour change models

Data concerning the theories employed was identified within the included papers using the original reviews’ own definitions or conceptualisations of theory. That is, if an included review referenced a particular theory or model, it was subsequently included in our synthesis. Reference to theory was either found in specific citations of theories used by individual papers incorporated within the review (commonly included in summary tables within the included papers), or as a broader framework used by included reviews to collate and synthesise the identified literature. Key outcomes and conclusions regarding the utility of behaviour change models were identified similarly, using review authors’ references to the theories they cite within their Results and Discussion sections.

Data from the included studies were synthesised to identify: a) the behaviour change theories most commonly employed to understand and influence protective health behaviours during public health emergencies and infectious disease outbreaks and, b) any (in) consistency in the reported utility of different behaviour change theories.

## Results

### Original study selection

**Fig. 1:**
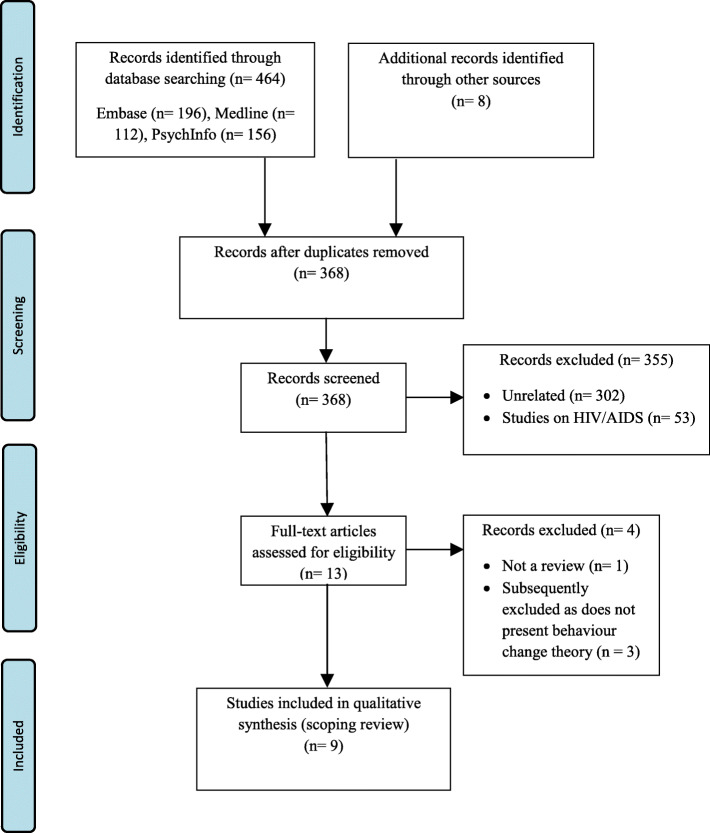
Flow diagram of the original selection process. PRISMA 2009 Flow Diagram adapted from: Moher, Liberati, Tetzlaff & Altman (2009) [49]

A total of 464 records were identified through database searching with an additional eight articles identified through other sources. Following the removal of duplicate citations, 368 papers were subjected to title/ abstract screening. Thirteen papers were retained for full-text eligibility assessment by the first and second authors. Following this assessment, one paper was excluded, leaving 12 remaining papers [37–48] (see Fig. 1). During the conduct of this review, the focus evolved to be explicitly concerned with only the application of theories rather than a broader focus on theoretically-related constructs. Subsequent reconsideration by the first author therefore led to three of these papers being excluded [42, 43, 47], as although they all presented constructs that are represented within behaviour change theories, none explicitly referenced theory. These papers are explicitly referenced here to signpost the interested reader to their existence.

Information concerning: (a) the article characteristics, (b) the application/ use of psychological theory within these reviews, (c) the total number of unique articles employing each behaviour change theory and, (d) a summary of the key conclusions regarding the utility of theory within each review, is collated and summarised in this Results section. 

### Top-up study selection

**Fig. 2:**
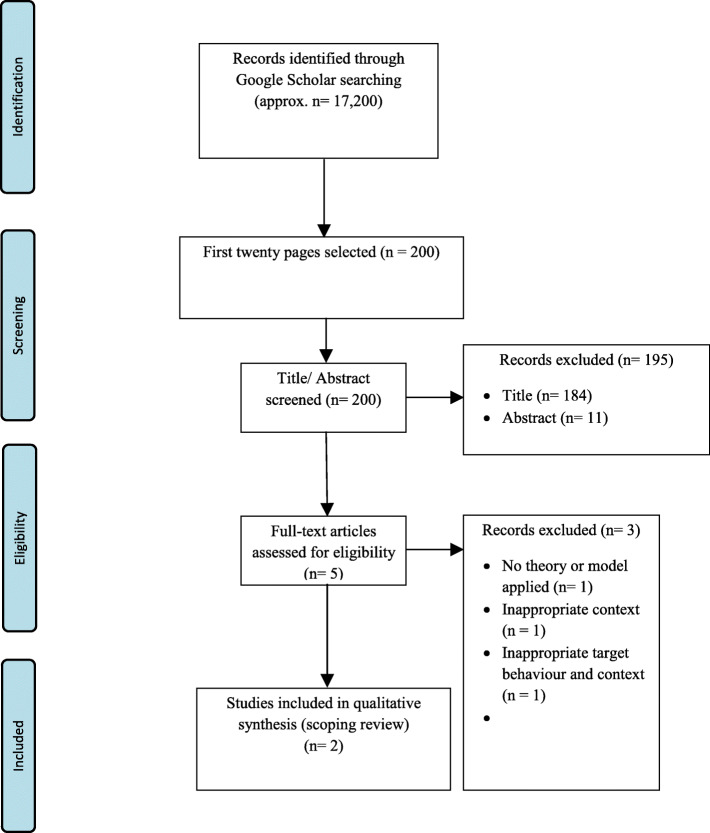
Flow diagram of the top-up study selection process. PRISMA 2009 Flow Diagram adapted from: Moher, Liberati, Tetzlaff & Altman (2009) [49]

A total of approximately 17,200 papers were identified using the Google Scholar search. Of these 17,200, the first 20 pages (200 hits) were subjected to title screening. Following this stage 16 hits were retained for abstract screening, which yielded five papers for full-text review. Following full-text review by the first and second author, two papers were retained for inclusion in this review [50, 51] (see Fig. 2). These two papers were subsequently incorporated into a revision of the initial synthesis and analysis and are presented in Table 1 and the Supplementary Information alongside literature identified through the original screening process.

**Table 1:** Reviews of theory use in the context of emergencies and outbreaks

Study	Papers (n)	Behaviour	Psychological Theory (times cited by studies included in the individual reviews)
Angus, Cairns, Purves, Bryce, MacDonald & Gordon (2013) [37]	61	Vaccination; hand hygiene; sharing injecting equipment; adherence to medication; reducing prescription and use of antibiotics; respiratory hygiene; safe sex; health screening uptake; drug injection behaviour; avoiding/ removing ticks; identifying Lyme disease; communicable disease prevention and control	Health Belief Model (15) Theory of Planned Behaviour (4) Social Cognitive Theory (Social Learning Theory) (4) Stage of Change (transtheoretical model) (8) Theory of Reasoned Action (1) Diffusion of Innovations (4) PRECEDE-PROCEED (3) Community Organization Locality Development Model (1) Precaution Adoption Process Model (1) Health Belief Model, Integrated Behavioural Model (1) Self-Regulation Model (1) New Comprehensive Health Seeking and Coping Paradigm based on Transactional Stress and Coping Model, Other (Health Seeking Paradigm) (1) Social Ecological Model (1). Health Belief Model, Theory of Reasoned Action, Social Cognitive Theory (1) Health Belief Model, Theory of Reasoned Action, Theory of Planned Behaviour, Social Cognitive Theory (1) Health Belief Model, Theory of Reasoned Action (1) Health Belief Model, Theory of Planned Behaviour (3) Health Belief Model, Social Cognitive Theory (2) Theory of Planned Behaviour, Social Cognitive Theory (1) Health Belief Model, Social Cognitive Theory, Diffusion of innovations, Other (unspecified) (1) Social Cognitive Theory, Behavioural Ecological Model (1) Social Cognitive Theory, Other (team-based learning theory and Information-Motivation Behavioural Skills (IMB) model) (2) Theory of Planned Behaviour, Extended Parallel Process Model (1) Theory of Reasoned Action, Social Cognitive Theory, Other (self-efficacy theory)^b^ (1) Health Belief Model, Theory of Reasoned Action, Theory of Planned Behaviour, PRECEDE-PROCEED, New model (1)
Bish & Michie (2010) [38]	26	Pandemic influenza vaccination intentions and behaviour	The majority of the studies (20) did not provide the theoretical model. Subjective expected utility theory (1) Health Belief Model (3) Health Belief Model, Theory of Planned Behaviour, Social Cognitive Theory (1) Health Belief Model, Theory of Planned Behaviour (1)
Bish & Michie (2011) [39]	12	Pandemic influenza vaccination intentions and behaviour	The majority of the studies (10) did not provide the theoretical model. Protection Motivation Theory (1) New model proposed by authors (1)
Bish, Michie, & Yardley (2011) [40]	25^a^	H1N1 influenza vaccination intentions and behaviour	The majority of the studies (20) did not provide the theoretical model used Health Belief Model, Protection Motivation Theory (1) Health Belief Model (2) Self-Regulation Model, Health Belief Model, Illness perceptions (1)
Bish, Yardley, Nicoll & Michie (2011) [41]	37	H1N1 influenza vaccination intentions and behaviour	The majority (32) of studies did not provide the theoretical model used Health Belief Model, Protection Motivation Theory (1) Health Belief Model (2) Self-Regulation Model, Health Belief Model & illness perceptions (1) Extended Theory of Planned Behaviour (1)
Bults, Beaujean, Richardus & Voeten (2015) [44]	70	Perceptions and responses to the 2009 H1N1 pandemic	Majority of studies (54) in the review did not cite the theory used. Health Belief Model (3) Health Belief Model, Protection Motivation Theory (1) Health Belief Model, Common Sense Model^c^ (1) Health Belief Model, Self-Regulation Model (1) Health Belief Model, Protection Motivation Theory, Extended Parallel Process Model (1) Health Belief Model, Theory of Planned Behaviour (1) Health Belief Model, Protection Motivation Theory, Theory of Reasoned Action, Theory of Planned Behaviour (1) Health Belief Model, Precaution Adoption Process Model (1) Protection Motivation Theory (1) Protection Motivation Theory, Trust and Confidence Model (1) Theory of Planned Behaviour (1) Risk Communication Framework (1) Social Ecological Model (1) Trust Determination Model (1)
Corace, Srigley, Hargadon, Yu, MacDonald, Fabrigar, & Garber (2016) [50]	10	Healthcare worker influenza vaccination	Health Belief Model (5) Theory of Planned Behaviour (2) Triandis Model of Interpersonal Behaviour (1) Health Belief Model, Behavioural Intention Model (1) Risk Perception Attitude (1)
Ejeta, Ardalan & Paton (2015) [45]	33	Outbreak preparedness (H1N1 vaccination; blood donation during influenza; non-pharmaceutical measures against influenza; health workers preparedness to respond to emergencies); flood preparedness; earthquake preparedness; climate change preparedness; tornado preparedness, preparedness for terrorism; preparedness for general emergency	Health Belief Model (4) Health Belief Model, Theory of Planned Behaviour (1) Health Belief Model, multidimensional locus of control theory (1) Extended Parallel Process Model (5) Theory of Planned Behaviour (3) Social Cognitive Model^d^ (3) Protection Motivation Theory (3) New model derived from existing models (Social Predictor Model of Intentions) (1) Vested interest theory (2) Protective Action Decision Model (1) Affective and Cognitive Route (1) Expectancy-valency models (1) Outcome expectancy and self- efficacy (1) Person-relative-to-Event model (1) Social Ecological Resilience theory (1) Precaution Adoption Theory (1) Theory of Communicating Actionable Risk (1) Adaptation terror preparedness model (1) Transtheoretical Model (1)
Leppin & Aro (2009) [46]	30	Perceived risk of SARS and avian influenza; Protective behaviours (e.g., hand washing, diet, exercise, wearing face masks)	Majority of studies (19) in the review did not cite the theory used. Health Belief Model (4) Health Belief Model, Theory of Reasoned Action, Theory of Planned Behaviour, Self-Efficacy Theory (1) Health Belief Model, Theory of Planned Behaviour (1) Health Belief Model, Protection Motivation Theory (1) Protection Motivation Theory (1) Precaution Adoption Model (1) State-dependent expected utility framework (1) Theory of optimistic bias/ Unrealistic optimism (1)
Omori, Kuligowski, Butler, & Gwynne (2017)^e^ [52]	N/A – Not systematic review	Emergency response within buildings (e.g., evacuation)	Doesn’t list theories used by papers. Uses the Protective Action Decision Model as a framework for the review.
Prematunge, Corace, McCarthy, Nair, Pugsley & Garber (2012) [48]	20	Uptake or refusal of PH1N1 vaccine	Doesn’t list theories used by cited papers. Health Belief Model is used as a conceptual model to present analyses.
Schmid, Rauber, Betsch, Lidolt & Denker (2017) [51]	470	Influenza vaccination hesitancy	Doesn’t list theories used by cited papers. Theory of Planned Behaviour used as a conceptual framework. Outcomes discussed in relation to 4C model of reasons for non-vaccination
Westcott, Ronan, Bambrick, & Taylor (2017)^e^ [53]	N/A – not systematic review	Bushfire preparedness among animal owners	Doesn’t list theories used by papers. Protection Motivation Theory used as framework for the review.

^a^Only 24 papers were included in the authors’ review summary table, thus reflecting the total number of papers listed in our psychological theory column

^b^Self-efficacy theory is listed as ‘other’ within this paper, but we have incorporated this within our review alongside the additional, explicit self-efficacy theory citation from another included review paper

^c^Although this is presented as the Common Sense Model, distinct from the other Self-Regulation Model citation within Bults and colleagues’ review, further examination of the original papers reveals they are based on the same underlying model, and so are integrated in our synthesis

^d^Examination of the reviews citing Social Cognitive Theory [37, 38] and the Social Cognitive Model [45] has revealed that these are distinct theories and are therefore included in our synthesis as such

^e^The authors of these reviews briefly cite examples of additional theories before settling on the Protection Motivation Theory and the Protective Action Decision Model respectively. Only these two theories are included in this table and in our synthesis

### Optimised top-up study selection

**Fig. 3:**
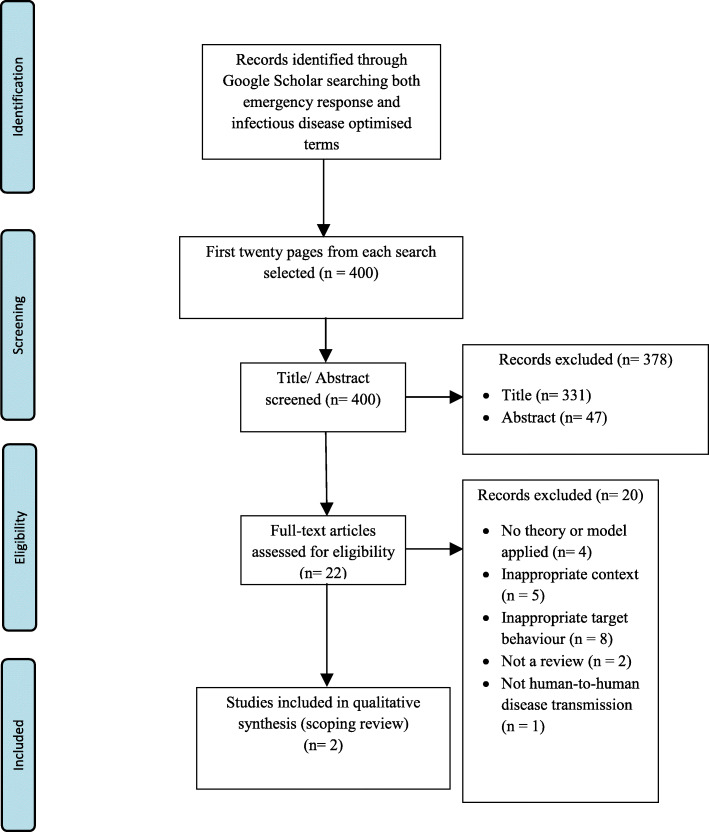
Flow diagram of the optimised top-up study selection process. PRISMA 2009 Flow Diagram adapted from: Moher, Liberati, Tetzlaff & Altman (2009) [49]

The first 20 pages of each optimised top-up search were subjected to title screening (400 hits in total). Following this stage, 47 hits were retained for abstract screening, which yielded 22 papers for full-text review. Following full-text review by the first author, two papers were retained for inclusion in this review [52, 53] (see Fig. 3).

As part of the full-text review process, the decision was taken to exclude two reviews [54, 55] which did examine the application of theory in a similar context to that of the two included reviews [52, 53]. This decision was taken as the focus of these reviews were more on understanding behaviour during an emergency, rather than the primarily protective or preventative focus of this review. These citations are presented here, in order to signpost them to interested readers.

As for the initial top-up process, the two included papers were incorporated into a revision of the synthesis and analysis presented within this manuscript and the accompanying Supplementary Information.

The synthesis including both original and all top-up studies is presented together in the following sections.

### Article characteristics

In addition to peer reviewed academic publications, the sample included reports published by the Department of Health, UK [39, 40] and the European Centre for Disease Prevention and Control [37], and one review from within an unpublished doctoral thesis [44]^1^. Six of the reviews cited in this synthesis had at least one author in common with another review cited herein [38–41, 48, 50], with one [39] explicitly cited as an update of another [38]. To examine the extent to which the papers included in our review were sampling the same citations, we looked across all papers to see how many citations were fully independent (that is, not cited in any other review included in this manuscript). To do this we either: a) examined the list of included studies provided by the authors of each systematic review, or; b) where such a list was not provided, we examined the full reference list for the manuscript. Although the percentage of unique papers varied substantially from review to review, each paper had an average of 66.9% unique papers (see Table 2 for the full breakdown).

**Table 2:** Unique citations across all papers included in the review

Author	# of papers	# unique	% unique
Angus, Cairns, Purves, Bryce, MacDonald & Gordon (2013) [37]	61	60	98.4
Bish & Michie (2010) [38]	26	8	30.8
Bish & Michie (2011) [39]	12	8	66.7
Bish, Michie, & Yardley (2011) [40]	24	3	12.5
Bish, Yardley, Nicoll & Michie (2011) [41]	37	6	16.2
Bults, Beaujean, Richardus & Voeten (2015) [44]	70	34	48.6
Corace, Srigley, Hargadon, Yu, MacDonald, Fabrigar, & Garber (2016)	10	9	90.0
Ejeta, Ardalan & Paton (2015) [45]	33	32	97.0
Leppin & Aro (2009) [46]	30	17	56.7
Omori, Kuligowski, Butler, & Gwynne (2017)^a^ [52]	58	57	98.3
Prematunge, Corace, McCarthy, Nair, Pugsley & Garber (2012) [48]	20	12	60.0
Schmid, Rauber, Betsch, Lidolt & Denker (2017)^b^ [51]	508	485	95.5
Westcott, Ronan, Bambrick, & Taylor (2017)^a^ [53]	101	101	100.0
**AVERAGE**			**66.9**

^a^As this was not a systematic review, the full reference list was searched

^b^Although the authors indicate that 470 papers were included in the review, no list of these papers was provided. Given this, the full reference list of 508 citations was searched

Although heavily focused on H1N1 pandemic influenza and vaccination behaviour, these papers did cover a wide range of health-related behaviours (e.g., hand hygiene, face mask wearing) across various infectious disease and public health emergency contexts (e.g., natural disasters, terrorism). Specifically, 10 papers [37–41, 44, 45, 48, 50, 51] looked at uptake of vaccination against influenza (primarily pandemic, but also including seasonal). One paper [44] considered the relationship between risk perception and preventive behaviour related to SARS and avian influenza (e.g., hand washing, diet, exercise, wearing face masks). Four papers [37, 44–46] also considered other outbreak preparedness behaviours in addition to vaccination (e.g. hand hygiene, non-pharmaceutical measures against influenza etc.). Three papers [45, 52, 53] investigated the application of theories/model in non-infectious disease emergencies and disasters (e.g., flood disaster preparedness, earthquake preparedness, climate change, fire preparedness, bushfire emergencies, tornado preparedness, & terrorism preparedness). All reviews were published between 2009 [46] and 2017 [51–53] and all except two [52, 53] employed a systematic approach to data collection.

### Frequency of theory application

In the first instance, we looked across the 13 review papers to see which theories were cited by the highest number of reviews (regardless of the number of cited papers using each theory within each review, and incorporating mentions of particular theories as frameworks for synthesis as in [48, 51–53]). This initial examination revealed that the Health Belief Model [56] was explicitly represented in the most review papers (nine – [37, 38, 40, 41, 44–46, 48, 50]), followed by the Theory of Planned Behaviour [57] (eight – [37, 38, 41, 44–46, 50, 51]) and Protection Motivation Theory [58] (seven – [39–41, 44–46, 53]), Precaution Adoption Process Model [59] (four – [37, 44–46]), and the Common Sense Model of Self-Regulation [60] (four – [37, 40, 41, 44]). A further two models were each included in three review papers: Extended Parallel Process Model [61] (three – [37, 44, 45]), and the Theory of Reasoned Action [62] (three – [37, 44, 46]). All other models were cited two times or fewer (see Table 1)^2^.

Next, we collated and examined the papers cited across all reviews to identify the most frequently cited theories overall. Articles that the reviews specifically cited as including behavioural theories were collated from the nine reviews that extracted such data [37–41, 44–46, 50]). Thus, papers were not included either: a) if the reviews did not indicate that such papers included behavioural theories or b) from the reviews that did not provide detail on the theories used by their cited papers (specifically, [48, 51–53]). Papers that were listed as including behavioural theory and were cited by multiple reviews (*n* = 9) were only included once, leaving a total of 137 unique papers which were listed by the various review authors as incorporating one or more behaviour change theories. See Supplementary Information 3 for an overview of which theories were cited in multiple reviews, and for the overall number of times each theory was cited in a unique paper across all reviews.

Across the cited literature, four behaviour change theories were applied more than 10 times. These were: Health Belief Model (60 times), Theory of Planned Behaviour (22 times), Social Cognitive Theory [63] (15 times) and Protection Motivation Theory (12 times). Other theories that were applied between four and 10 times include: Trans-Theoretical Model [64] (9 times), Theory of Reasoned Action (8 times), Extended Parallel Process Model (7 times), Diffusion of Innovation [65] (5 times), Precaution Adoption Process Model (4 times), and PRECEDE-PROCEED (which stands for Predisposing, Reinforcing and Enabling Constructs in Educational Diagnosis and Evaluation – Policy Regulatory and Organisational Constructs in Educational and Environmental Development) [66] (4 times). All other theories were applied fewer than four times.

### Utility of theories

When considering the application of behaviour change theories for research, the included reviews report mixed success. While all of the papers cited by [37] were informed by theory, several other authors reported that relatively few papers included within their reviews explicitly use behaviour change theories [40, 41, 44, 46, 50]. For example, one review found that only around one third of cited papers explicitly refer to a theoretical model [46] and another failed to find any interventions that used behavioural frameworks in their development [50].

Nevertheless, where theory was cited, across reviews there was support for the utility of the most cited theories across reviews and within individual papers. Particular support was provided in the key outcomes and conclusions across reviews for: Health Belief Model [37–40, 45, 46, 48, 50], the Theory of Planned Behaviour [37–40, 45, 50, 51], Protection Motivation Theory [38–41, 44, 53], and the Common-Sense Model [38–40] (see Supplementary Information 2 for a summary of theory-related outcomes for each review cited herein). Although this is based on key outcomes/ conclusions and not an exhaustive list of all successful theories reported within/across reviews, the commonly applied behaviour change theories do seem to be identified as relevant for understanding and explaining human behaviour within an infectious disease and emergency response context.

However, the use of these theories was not universally lauded: for example, one review argues that the Health Belief Model, Protection Motivation Theory and the Theory of Planned Behaviour do not adequately allow for emotional factors in behavioural decision making (of the three, Protection Motivation Theory does incorporate fear, however the impact of emotion is on threat-appraisals rather than a direct effect of emotion on action [22]) [38]. Indeed, one review exploring the application of Protection Motivation Theory for animal owners and emergency responders in the context of bushfire emergencies suggests that emotional attachment (to animals) could override adaptive responding [53]. Similarly, one review strongly supports the relevance of the Extended Parallel Process Model for disaster and emergency preparedness but draws on a study examining the mediating role of fear on the threat – preparedness relationship to argue for further work applying the Extended Parallel Process Model to look beyond just threat and efficacy in their applications [45]. Furthermore, although several papers do support the relevance of the Theory of Planned Behaviour within this context (e.g., [37, 38, 51]), one review does identify inconsistent findings regarding the role of the Theory of Planned Behaviour for predicting behaviour within their cited papers (but still advocates the relevance of this theory for vaccination behaviour) [50].

In terms of intervention development, one review concludes that although there is clear evidence for the success of theory-based interventions for communicable disease control and prevention, there is no substantive difference in theories informing effective or ineffective interventions [37]. Rather than specific theories being of critical importance for intervention development, it is instead the *role* of theory that is important; positive effects were reported where theories were used to design and develop interventions, but more mixed effects were reported when theories were only used to *evaluate* interventions [37]. This echoes points made by Leppin and Aro in their review of risk perceptions in relation to Severe Acute Respiratory Syndrome (SARS) and avian influenza. Specifically: 1) few studies explicitly or theoretically define risk perceptions, 2) there is a disproportionate focus on risk probability over risk severity and, 3) there is a need for further work to empirically examine the role of risk perceptions as represented within multifactor models rather than just through bivariate relationships [46].

Overall, this literature synthesis yields two key conclusions. Firstly, behaviour change theories are of clear relevance for understanding behaviour in the context of infectious diseases and emergency response. Secondly, and related to the first conclusion, there is a definite requirement for further work to systematically examine, incorporate and test full behaviour change models within research and interventions in the context of infectious disease and emergency response.

### Summary

Papers incorporating a total of 44 different theories (in various combinations) were collated across these reviews.^3^ The Health Belief Model, Theory of Planned Behaviour, and Protection Motivation Theory were the most cited theories both across reviews and by individual papers included within reviews. Other theories that were commonly cited include: Precaution Adoption Process Model, Extended Parallel Process Model, Theory of Reasoned Action, and Social Cognitive Theory.

Following a synthesis of the key theory-related outcomes and conclusions, there was broad support for the applicability of the most commonly cited theories (listed above) within this context. However, despite this broad support for the applicability of these theories, several reviews reported low levels of explicit use of behaviour change theories in the research they cited.

Taken together, these results suggest that the most commonly cited theories reported herein represent an excellent starting point for practitioners and public health professionals looking to model and enact behaviour change in the context of infectious disease and emergency response. This point is explored in more detail in the Recommendations subsection of the Discussion.

## Discussion

To the best of the authors’ knowledge, this scoping review represents the first attempt to systematically collate review data concerning the application of behaviour change theories within a broad infectious disease and emergency response context. In this review we have synthesised the health behaviours, theories, and applications presented across 13 review papers drawn from both peer reviewed journals and grey literature in the context of infectious disease and emergency response. This synthesis enables us to provide some key recommendations and suggestions for infectious disease modellers, researchers, and public health professionals looking to apply behaviour change theory to understand and influence behaviour in this context.

Looking across the 13 reviews included in our synthesis provides a clear picture of the typical use of behaviour change theories across the current context. Firstly, many papers included within these reviews do not seem to be explicitly based on a specific theory of behaviour change. Secondly, whether we take a high-level approach (i.e., number of theories cited by multiple reviews) or a more granular approach (i.e., number of citations per theory across all reviews) to the synthesis, the conclusions are broadly the same. As per Michie and colleagues, only a small number of theories were most commonly cited despite the broad number of theories available (83 theories detailed within [22], and 44 distinct theories cited at least once within our included review papers, although these may not all be present in Michie and colleagues work [22]). Specifically, three theories stand out as the most commonly applied: Health Belief Model, Theory of Planned Behaviour, and Protection Motivation Theory. Another four theories are also repeatedly, but less frequently, cited: Precaution Adoption Process Model, Extended Parallel Process Model, Theory of Reasoned Action, and Social Cognitive Theory.

Of these seven theories, four are consistent with the most frequently used theories as identified by Michie and colleagues [22] (Theory of Planned Behaviour, Health Belief Model, Precaution Adoption Process Model, & Social Cognitive Theory), and with some of the theories cited as frequently used across other health behaviour contexts e.g., [27, 28] (Health Belief Model, Theory of Planned Behaviour, & Social Cognitive Theory). Of the remaining theories, one (Theory of Reasoned Action) is closely linked to the Theory of Planned Behaviour (the latter having developed from the former [57]). The final two most cited theories – Protection Motivation Theory and the Extended Parallel Process Model —are closely related (the latter builds on the former [61]) and both are particularly concerned with the processes underlying fear appeals and threat messaging, a focus with clear relevance in the context of this review.

Having identified the commonly applied theories, we subsequently conducted a rapid synthesis of the included reviews’ key outcomes with regards to the utility of behaviour change theory. On the basis of this synthesis, we make two key conclusions. Firstly, the most frequently cited theories do find broad (though not universal) support within the cited literature. Secondly, despite this broad applicability, several reviews cited herein highlight the relative absence of behaviour change theory within research conducted in this context [40, 41, 44, 46, 50]. Indeed, several of the reviews included herein explicitly advocate further work to study the thorough application of both individual theories and a range of multivariable theories, considering the interrelationship between model factors/ components (e.g., [37, 38, 40, 41, 44–46, 50]).

Overall, a clear take-home message from the current review is that there are a range of commonly applied behaviour change theories with broad support for their use within an infectious disease and emergency response context. In the following section, these findings are used to form the basis for recommendations concerning the use of behaviour change theory by researchers, practitioners, and modellers in both research and practice.

### Recommendations

Based on the results of our synthesis, there are two broad categories of recommendations for future work applying and incorporating behaviour change theories into both: 1) research and practice (i.e., understanding behaviour, and developing & deploying effective interventions), and; 2) infectious disease modelling.

Furthermore, although most of the literature screening and synthesis for this review was conducted prior to the COVID-19 pandemic, we are aware of some recent and relevant work detailing recommendations and guidance for the use of behavioural science to tackle COVID-19 in practice. Some of this work has also been incorporated into this section to signpost interested readers to additional material of relevance.

#### Researchers and practitioners

Behavioural science can play a critical role in combatting the effects of a global pandemic, such as COVID-19, both through informing our understanding of public perceptions of the virus, and through developing interventions to reduce barriers and facilitate uptake of recommended behaviours [13]. Indeed, in the context of COVID-19, West and colleagues indicate that in the absence of robust intervention data, behaviour change theories and constructs should be used to inform the development of policy and practice for increasing uptake of self-protective behaviours [67].

Unfortunately, however, multiple reviews cited herein lament the absence of behaviour change theory in work conducted to date [40, 41, 44, 46, 50], and advocate for the more in-depth study of various behaviour change theory within this context [37, 38, 40, 41, 44–46, 50]. Our primary recommendation therefore reinforces that advocated in the literature cited herein. Specifically, we recommend that researchers and practitioners working in the context of infectious disease and emergency response, should: a) draw on the available theoretical literature and; b) work with experts in behavioural science to inform both empirical work to understand behaviour, and the design and implementation of interventions to affect behaviour. This primary recommendation is supported by several additional recommendations, which are unpacked in the subsequent paragraphs.

In describing the way forward for behaviour change theorising, Michie and colleagues [22] note that the most popular behaviour change theories are relatively context agnostic, and that there may be important insights to be taken from models that are more context dependent. In order to enhance the translation of research into public health practice, we therefore recommend that future research and intervention development should consider both general theories and theories that most closely fits the context of study. The theories identified within this review represent a mix of both context agnostic (e.g., Health Belief Model, Theory of Planned Behaviour) and some more context specific (e.g., Protection Motivation Theory and Extended Parallel Process Model) theories, and would therefore seem to be a good starting point for researchers and practitioners working in this area. Furthermore, although not a theory identified within the current review (see Limitations section), the COM-B (capability, opportunity, motivation and behaviour) model [68] has been advocated as a key starting point for interventions to reduce the transmission of Severe Acute Respiratory Syndrome Coronavirus 2 (SARS-CoV-2) during the COVID-19 pandemic (e.g., [67]). We would therefore further recommend considering the application of this theory when either conducting research or delivering research into practice.

While these recommendations are consistent with the Medical Council Guidelines [21], and echo recent work by Michie and colleagues (e.g., [22, 23]), it is critically important to approach the incorporation of behaviour change theory –particularly within intervention design—in a systematic fashion [23]. Indeed, one review included within our synthesis found that theory had a positive impact on the success of interventions when used at the design and development stage, relative to theory used only at the evaluation stage [37]. To facilitate the systematic and appropriate use of behaviour change theory, we therefore strongly recommend that researchers and practitioners involve expert behavioural scientists in the design and implementation process, and also make use of available guidance within the behaviour change literature. For example, we are aware of a thorough guide to designing interventions using the Behaviour Change Wheel [23] that may be of use to practitioners.

We also echo Michie and Prestwich’s recommendation for researchers to use their Theory Coding Scheme (a list of 19 items for coding the use of theory within intervention design) to both: a) allow for researchers and practitioners to systematically assess the incorporation of theory within existing interventions and, b) facilitate transparent reporting of the incorporation of theory within novel interventions [69]. Using these resources will enable researchers and practitioners to overcome the limited use of theory acknowledged by the papers included in this review [40, 41, 44, 46, 50] while still avoiding “It Seemed Like A Good Idea At The Time” ([23], p14) interventions.

Lastly, we include a note on the role of behavioural science in combatting the COVID-19 pandemic. Helpfully, a range of prominent behavioural scientists have developed guidelines and recommendations for the application of behavioural science within the context of COVID-19. For example, Michie and colleagues advocate four principles to help reduce transmission by effecting behaviour change [70]; the British Psychological Society have compiled a list of nine recommendations to optimise the effectiveness of changing policy and communication/ guidance [71], and; West et al. draw upon COM-B to provide an account of components that need to be addressed in order to increase uptake of specific COVID-19 transmission-reduction behaviours [67]. While this is by no means an exhaustive list, it does reinforce the importance of explicitly and systematically incorporating behavioural science into research and practice within the context of infectious disease and emergency response. Alongside our recommendations detailed above, we therefore further advocate that interested readers explore these principles and guidelines in more detail.

#### Mathematical Modellers

In our sister review [26] we find that although the ‘gold standard’ for incorporating protective behaviour into infectious disease modelling involves the incorporation of a range of cognitive and social constructs, there is very little explicit reference to well-recognised theories of behaviour change (indeed, only five of 42 included papers make any such reference [26]). Acknowledging the necessary trade-off between accurately modelling human behaviour and the computational demands of such modelling [72], Weston and colleagues echo previous recommendations for modellers to familiarise themselves with the relevant behaviour change literature in order to improve their awareness of the main factors underlying human behaviour [24, 73]. As a result, the recommendation was made for infectious disease modellers to closely consult with the psychological literature concerning the predictors of health behaviour when developing their models [26].

Based on the outcomes of this current review, we can provide further guidance for infectious disease modellers on both where to begin with this familiarisation, and how/ where to involve behavioural scientists in the process. Firstly, in both the current review and Weston and colleagues work, the Health Belief Model is the most commonly cited behaviour change theory. We therefore agree that the Health Belief Model represents an appropriate base on which to build infectious disease models incorporating human behaviour [26]. Nevertheless, as noted in the modelling review, there are a broad range of additional factors—including emotional and social constructs—that should be more fully considered when representing infection prevention behaviour [26].

Given the emphasis on Protection Motivation Theory and the Extended Parallel Process Model within the literature reported in the current review, we first suggest that infectious disease modellers should consider these models alongside the Health Belief Model to help improve the modelling of emotional responding and defensive avoidance behaviour (but see also Bish & Michie for further recommendations concerning the use of parallel processing models to incorporate cognitive and emotional constructs, [38]). Similarly, we would also recommend infectious disease modellers use Social Cognitive Theory, identified as a prominent public health behaviour change theory in the current review, as another starting point for behavioural model formulation. By more fully considering these theories and associated literature, infectious disease modellers will be well prepared to accurately and precisely model a range of relevant social, cognitive, and emotional constructs that may be associated with behavioural responses to a public health emergency.

Although this familiarisation exercise will be invaluable in helping modellers to develop a deeper understanding of the factors underlying behaviour change and is consistent with recommendations from previous literature as outlined above, it is pertinent to echo a recommendation made by Michie and colleagues in the context of intervention design. That is, it is important to ensure that the theory selected is appropriate for the type of behaviour in question. For example, if a behaviour is more likely to be influenced by habitual factors then models concerning deliberative and reflective processing are less likely to be relevant [22]. As this current review has focused on identifying the broad state of the art for incorporating behaviour change theory within infectious disease and emergency contexts, a full consideration of the appropriate theories for each individual behaviour in each specific disease or emergency context is unfortunately outside scope. However, we recommend that infectious disease modellers work closely with behavioural scientists in the design and development of their models to ensure that the most appropriate theories are being consulted and incorporated for a given target behaviour or context. Through this greater collaboration between modellers and behavioural scientists, the discipline will be able to develop a more in-depth understanding of the requirements for behavioural theory and the computational limitations to their incorporation within infectious disease models.

### Limitations and future research

Although the review reported herein represents an impressive effort at addressing a herculean task (namely, the collation of psychological behaviour change theories applied across infectious disease and emergency response contexts), as with any large-scale review there are inevitably trade-offs and potential limitations that should be considered when interpreting the outcomes and recommendations from this review.

Firstly, although other emergency contexts are represented within the current review, we acknowledge that the papers included within this review are predominantly focused on infectious diseases. Although the search strategy did include terms relating to other specific civil emergencies (e.g., terrorism), and emergencies generally (e.g., emergency response, emergency resilience), there were more search terms relating to infectious disease outbreaks/ pandemics. We therefore recommend that future reviews in this area should utilise search strategies optimised more clearly to reflect the full breadth of public health emergency contexts.

Secondly, and similarly to the first limitation, we are aware of some prominent behaviour change theories (e.g., COM-B) that are not represented in the reviews cited herein. Although this may represent a limitation of our search strategy, generic phrases and subject headings relating to theoretical models were included to ensure a breadth of focus. Furthermore, as our focus was on identifying review articles that have themselves collated primary research involving behaviour change theories in the context of infectious disease and emergency response, it follows that any prominently applied theories should have also been represented within our sample regardless of the specific search terms we used. We are therefore confident that the theories identified herein represent an accurate overview of the most commonly cited theories within this specific context over the period in question (i.e., pre COVID-19).

Thirdly, we acknowledge that several of the reviews included herein were authored by some of the same individuals [38–41, 48, 50], with at least one [39] explicitly cited as an update of another [38]. Although this may influence the macro-representation of theories across reviews (i.e., at review-level), our decision to also examine the frequency of theory use at a micro-level (i.e., at individual cited study level, excluding repeat citations across reviews) with similar results mitigates the likely impact of this.

Nevertheless, we acknowledge that frequency of theory use – a key outcome within the current review – is not the same thing as contextual-relevance of theory within these contexts. However, the purpose of this review was to identify the most commonly employed theories of behaviour change within the infectious disease and emergency response contexts. Given this focus, we believe that the emphasis on theory frequency within the current review is well founded. Nevertheless, we do also provide a synthesis and summary of the key outcomes and conclusions in order to further guide researchers and mathematical modellers to the points of commonality and divergence within the extant review literature. We hope that this review will therefore serve as a jumping off point for further research and modelling work building on our outcomes as detailed in the preceding Recommendations section.

Finally, given the breadth of available primary literature, the proliferation of available reviews of behaviour change theories, and the specificity of our research question (i.e., to identify commonly applied theories of behaviour change within a public health emergency context) we elected to conduct a review of reviews rather than a systematic review of all literature. Similarly, although driven by pragmatic concerns, our decision to conduct our top up searches using the first 20 pages of Google Scholar searches may have limited the number of potentially relevant manuscripts for screening. Furthermore, given both the unclear relevance /lack of access to a number of papers within the current review, we elected to wholesale exclude reviews concerning sexually transmitted infection. Although these decisions and outcomes may have narrowed our focus, we argue that the close parallels between the theories identified herein and those remarked as commonplace within previous review work (e.g., [22]) render this claim ill founded. Indeed, when combined, our top-up searches (which were sorted by relevance) allowed us to search through 600 citations published since 2016. The number of unique citations across papers included in our review (see Table 2) further suggests that we have succeeded in drawing together a broader range of literature than the independent systematic reviews themselves managed.

Nevertheless, we consider the current work to be an initial attempt at identifying and integrating the literature applying behaviour change theories in the context of infectious disease and emergency response. We therefore invite and encourage the conduct of a full and systematic review of all primary literature concerning the application of behaviour change theories across public health emergency contexts using our search strategy and extraction terms as a guide.

## Conclusion

Behavioural science can play a critical role in combatting the effects of an infectious disease outbreak or public health emergency, such as the COVID-19 pandemic. However, the proliferation of available theories, with either general or specific application, can made the landscape confusing for researchers, infectious disease modellers and public health practitioners alike. In an effort to simplify the considerable behaviour change literature for ease of use by public health emergency researchers, we conducted a systematised scoping ‘review of the reviews’ concerning the application of behaviour change theories in infectious disease and emergency response emergency contexts. Our search strategies revealed 13 relevant review papers from which we were able to identify and collate the seven most commonly cited and applied behaviour change theories in this context: Health Belief Model, Theory of Planned Behaviour, and Protection Motivation Theory as most commonly applied, followed by Precaution Adoption Process Model, Extended Parallel Process Model, Theory of Reasoned Action, and Social Cognitive Theory.

Following a synthesis of the key theory-related outcomes and conclusions, we conclude that while there is broad support for the use of the most commonly cited theories within this context, the previous application of these theories within the literature is patchy. That is, much research in this context has not drawn on relevant theories of behaviour change.

Based on these identified theories and our synthesis of review outcomes, and in conjunction with a recent review by Weston and colleagues [26], we make recommendations to assist researchers, intervention designers, and mathematical modellers to incorporate psychological behaviour change theories within infectious disease and emergency response contexts. First, we echo previous recommendations that future research and intervention design within this context should be based explicitly and systematically on relevant behaviour change theories, and in close consultation with experts in behavioural science. The theories identified herein represent an excellent starting point for this work, and we further signpost the reader to both general materials to aid in intervention design, and guiding principles for practitioners and researchers working on COVID-19. Second, we recommend that mathematical modellers should consult the theories identified herein, and work closely with behavioural scientists to familiarise themselves with the key factors underlying behaviour change within an infectious disease and emergency response context.

Considered together, the results and recommendations reported herein therefore represent an important resource to enable researchers, modellers, and practitioners working in the context of infectious disease and emergency response to better incorporate a systematic and evidence-based consideration of human behaviour into their work.

## Supplementary information

**Additional file 1: Supplementary Information 1.** Search terms for the original selection process

**Additional file 2: Supplementary Information 2.** Summary of key theory-related conclusions

**Additional file 3: Supplementary Information 3.** Breakdown of: a) how many/ which theories are presented across multiple reviews, and; b) how many times each theory is cited by a unique article across all reviews

## Data Availability

All data generated or analysed during this study are included in this published article and its supplementary information files.

## Abbreviations

COM-B: Capability, Opportunity, Motivation and Behaviour
COVID19: Coronavirus disease 2019
HIV: Human Immunodeficiency Virus
MeSH: Medical Subject Heading
PRECEDE-PROCEED: Predisposing, Reinforcing and Enabling Constructs in Educational Diagnosis and Evaluation – Policy Regulatory and Organisational Constructs in Educational and Environmental Development
SARS: Severe Acute Respiratory Syndrome
SARS-CoV-2: Severe Acute Respiratory Syndrome Coronavirus 2
STI: Sexually Transmitted Infection
UK: United Kingdom
WHO: World Health Organisation

## Footnotes

1 This review [44] forms part of a broader PhD thesis in which it is presented as a chapter. Given the focus of the chapter on conducting a systematic review of H1N1 perceptions and responses, and the consistency in the theories identified within the review chapter and the thesis’ introductory chapter, we elected to include only the specific review chapter in this synthesis.

2 Where there was close overlap between model/ theory names, we conducted a Google Search to determine whether the tiles likely reflect the same, or different, models. Consequently, Social Ecological Theory (see [37, 44]) and the Social Ecological Resilience Model [45] have been included as separate models in this synthesis. On closer inspection, Subjective Expected Utility Theory [38] and State Dependent Expected Utility Framework [46] refer to the same paper, and so are collapsed together herein. Precaution Adoption Theory [45] and Precaution Adoption Process Model [37, 44, 46] are interpreted as referring to the same theory and so are included together.

3 This number includes four models cited as ‘other’ within [37] and the four new models identified within [37, 39, 45], but does not include the 4Cs model described by [51] as this was used to discuss review outcomes within the review and was not listed as a theory cited by papers within the review.

## References

[1] Cabinet Office (2017). National risk register of civil emergencies 2017 edition.

[2] Johns Hopkins University & Medicine. COVID-19 Dashboard. https://coronavirus.jhu.edu/map.html. Accessed 8 August 2020.

[3] Centers for Disease Control and Prevention. Ebola (Ebola virus disease): 2014–2016 Ebola outbreak in West Africa. https://www.cdc.gov/vhf/ebola/history/2014-2016-outbreak/index.html. Accessed 20 Dec 2019.

[4] World Health Organisation. Ebola in the Democratic Republic of the Congo. Health emegency update. https://www.who.int/emergencies/diseases/ebola/drc-2019. Accessed 20 Dec 2019.

[5] World Health Organisation. Middle East respiratory syndrome coronavirus (MERS-CoV). https://www.who.int/emergencies/mers-cov/en/. Accessed 20 Dec 2019.

[6] Hine D (2010). The 2009 influenza pandemic. An independent review of the UK response to the 2009 influenza pandemic.

[7] Dawood FS, Iuliano AD, Reed C, Meltzer MI, Shay DK, Cheng P-Y (2012). Estimated global mortality associated with the first 12 months of 2009 pandemic influenza a H1N1 virus circulation: a modelling study. Lancet Infect Dis.

[8] Centers for Disease Control and Prevention. Influenza (flu). 2009 H1N1 pandemic (H1N1pdm09 virus). https://www.cdc.gov/flu/pandemic-resources/2009-h1n1-pandemic.html. Accessed 20 Dec 2019.

[9] Smith KF, Goldberg M, Rosenthal S, Carlson L, Chen J, Chen C (2014). Global rise in human infectious disease outbreaks. J R Soc Interface.

[10] Ritchie H, Hasell J, Appel C, Roser M. Terrorism. https://ourworldindata.org/terrorism. Accessed 20 Dec 2019.

[11] The United Nations Office for Disaster Risk Reduction, Centre for Research on the Epidemiology of Disasters (2015). The human cost of natural disasters: A global perspective.

[12] World Health Organization (2017). Communicating risk in public health emergencies: a WHO guideline for emergency risk communication (erc) policy and practice.

[13] Michie S, Rubin GJ, Amlôt R. Behavioural science must be at the heart of the public health response to covid-19. BMJ Opin. 2020; https://blogs.bmj.com/bmj/2020/02/28/behavioural-science-must-be-at-the-heart-of-the-public-health-response-to-covid-19/. Accessed 8 Aug 2020.

[14] Carter H, Drury J, Rubin GJ, Williams R, Amlôt R (2013). The effect of communication during mass decontamination. Disaster Prev Manag.

[15] Jefferds MD, Laserson K, Fry AM, Roy S, Hayslett J, Grummer-Strawn L (2002). Adherence to antimicrobial inhalational anthrax prophylaxis among postal workers, Washington, DC, 2001. Emerg Infect Dis.

[16] Rubin GJ, Amlôt R, Page L, Wessely S (2009). Public perceptions, anxiety, and behaviour change in relation to the swine flu outbreak: cross sectional telephone survey. BMJ..

[17] Williams L, Rasmussen S, Kleczkowski A, Maharaj S, Cairns N (2015). Protection motivation theory and social distancing behaviour in response to a simulated infectious disease epidemic. Psychol Health Med.

[18] Rubin GJ, Potts HW, Michie S (2011). Likely uptake of swine and seasonal flu vaccines among healthcare workers. A cross-sectional analysis of UK telephone survey data. Vaccine..

[19] Weston D, Blackburn R, Potts HW, Hayward AC (2017). Predictors of self and parental vaccination decisions in England during the 2009 H1N1 pandemic: analysis of the flu watch pandemic cohort data. Vaccine..

[20] Keeling M, Danon L (2009). Mathematical modelling of infectious diseases. Br Med Bull.

[21] Craig P, Dieppe P, Macintyre S, Michie S, Nazareth I, Petticrew M (2008). Developing and evaluating complex interventions: the new Medical Research Council guidance. BMJ..

[22] Michie S, West R, Campbell R, Brown J, Gainforth H (2014). ABC of behaviour change theories.

[23] Michie S, Atkins L, West R (2014). The behaviour change wheel.

[24] Frıas-Martınez E, Williamson G, Frıas-Martınez V (2011). Agent-based modelling of epidemic spreading using social networks and human mobility patterns. Proceedings of the 2011 IEEE third international conference on privacy, security, risk and trust and IEEE third international conference on social computing.

[25] Epstein JM, Parker J, Cummings D, Hammond RA (2008). Coupled contagion dynamics of fear and disease: mathematical and computational explorations. PLoS One.

[26] Weston D, Hauck K, Amlôt R (2018). Infection prevention behaviour and infectious disease modelling: a review of the literature and recommendations for the future. BMC Public Health.

[27] Davis R, Campbell R, Hildon Z, Hobbs L, Michie S (2015). Theories of behaviour and behaviour change across the social and behavioural sciences: a scoping review. Health Psychol Rev.

[28] Glanz K, Bishop DB (2010). The role of behavioral science theory in development and implementation of public health interventions. Annu Rev Public Health.

[29] Glanz K, Rimer BK, Viswanath K (2015). Health behavior and health education: theory, research, and practice.

[30] Painter JE, Borba CP, Hynes M, Mays D, Glanz K (2008). The use of theory in health behavior research from 2000 to 2005: a systematic review. Ann Behav Med.

[31] Hovell MF, Hillman ER, Blumberg E, Sipan C, Atkins C, Hofstetter CR (1994). A behavioral-ecological model of adolescent sexual development: a template for aids prevention. J Sex Res.

[32] Wagenaar AC, Perry CL (1994). Community strategies for the reduction of youth drinking: theory and application. J Res Adolesc.

[33] Alfonzo MA (2005). To walk or not to walk? The hierarchy of walking needs. Environ Behav.

[34] Smith V, Devane D, Begley CM, Clarke M (2011). Methodology in conducting a systematic review of systematic reviews of healthcare interventions. BMC Med Res Methodol.

[35] Becker L, Oxman A, Higgins J, Green S (2008). Overview of reviews. Cochrane handbook for systematic reviews of interventions.

[36] Arksey H, O'Malley L (2005). Scoping studies: towards a methodological framework. Int J Soc Res Methodol.

[37] Angus K, Cairns G, Purves R, Bryce S, MacDonald L, Gordon R (2013). Systematic literature review to examine the evidence for the effectiveness of interventions that use theories and models of behaviour change: towards the prevention and control of communicable diseases.

[38] Bish A, Michie S (2010). Demographic and attitudinal determinants of protective behaviours during a pandemic: a review. Br J Health Psychol.

[39] Bish A, Michie S (2011). Demographic and attitudinal determinants of protective behaviours during a pandemic.

[40] Bish A, Michie S, Yardley L (2011). Factors associated with uptake of vaccination against pandemic influenza: scientific evidence base review.

[41] Bish A, Yardley L, Nicoll A, Michie S (2011). Factors associated with uptake of vaccination against pandemic influenza: a systematic review. Vaccine..

[42] Blasi F, Aliberti S, Mantero M, Centanni S (2012). Compliance with anti-H1N1 vaccine among healthcare workers and general population. Clin Microbiol Infect.

[43] Brien S, Kwong JC, Buckeridge DL (2012). The determinants of 2009 pandemic a/H1N1 influenza vaccination: a systematic review. Vaccine..

[44] Bults M, Beaujean DJMA, Richardus J, Voeten H, Bults M (2015). Perceptions and behavioural responses of the general public during the 2009 influenza A (H1N1) pandemic: a systematic review. Outbreaks of emerging infectious diseases: risk perceptions and behaviour of the general public (unpublished doctoral thesis).

[45] Ejeta LT, Ardalan A, Paton D. Application of behavioral theories to disaster and emergency health preparedness: a systematic review. PLoS Curr Disasters. 2015;7. 10.1371/currents.dis.31a8995ced321301466db400f1357829 PMC4494855 26203400

[46] Leppin A, Aro AR (2009). Risk perceptions related to SARS and avian influenza: theoretical foundations of current empirical research. Int J Behav Med.

[47] Nguyen T, Henningsen KH, Brehaut JC, Hoe E, Wilson K (2011). Acceptance of a pandemic influenza vaccine: a systematic review of surveys of the general public. Infect Drug Resist.

[48] Prematunge C, Corace K, McCarthy A, Nair RC, Pugsley R, Garber G (2012). Factors influencing pandemic influenza vaccination of healthcare workers—a systematic review. Vaccine..

[49] Moher D, Liberati A, Tetzlaff J, Altman DG, The PRISMA Group. Preferred reporting items for systematic reviews and meta21 analyses: the PRISMA statement. PLoS Med. 2009;6(7):e1000097. 10.1371/journal.pmed.1000097 PMC2707599 19621072

[50] Corace KM, Srigley JA, Hargadon DP, Yu D, MacDonald TK, Fabrigar LR (2016). Using behavior change frameworks to improve healthcare worker influenza vaccination rates: a systematic review. Vaccine..

[51] Schmid P, Rauber D, Betsch C, Lidolt G, Denker M-L (2017). Barriers of influenza vaccination intention and behavior–a systematic review of influenza vaccine hesitancy, 2005–2016. PLoS One.

[52] Omori H, Kuligowski ED, Gwynne SM, Butler KM (2017). Human response to emergency communication: a review of guidance on alerts and warning messages for emergencies in buildings. Fire Technol.

[53] Westcott R, Ronan K, Bambrick H, Taylor M (2017). Expanding protection motivation theory: investigating an application to animal owners and emergency responders in bushfire emergencies. BMC Psychology.

[54] Cheng Y, Liu D, Chen J, Namilae S, Thropp J, Seong Y. Human behavior under emergency and its simulation modeling: a review. In: Cassenti D, editor. Advances in human factors in simulation and modeling. AHFE 2018. Advances in intelligent systems and computing. 2019;780:313–325. https://doi.org/10.1007/978-3-319-94223-0_30.

[55] Drury J (2018). The role of social identity processes in mass emergency behaviour: an integrative review. Eur Rev Soc Psychol.

[56] Rosenstock IM (1974). Historical origins of the health belief model. Health Educ Monogr.

[57] Ajzen I (1991). The theory of planned behavior. Organ Behav Hum Decis Process.

[58] Rogers RW (1975). A protection motivation theory of fear appeals and attitude change. J Psychology.

[59] Weinstein ND, Sandman PM (1992). A model of the precaution adoption process: evidence from home radon testing. Health Psychol.

[60] Leventhal H, Brisette I, Leventhal EA, Cameron LD, Levanthal H (2003). The common-sense model of regulation of health and illness. The self-regulation of health and illness behaviour.

[61] Witte K (1992). Putting the fear back into fear appeals: the extended parallel process model. Commun Monogr.

[62] Fishbein M, Ajzen I. Belief, attitude, intention, and behaviour: an introduction to theory and research. Reading: Addison-Wesley; 1975.

[63] Bandura A (1986). Social foundations of thought and action: a social cognitive theory.

[64] Prochaska JO, DiClemente CC (1982). Transtheoretical therapy: toward a more integrative model of change. Psychotherapy.

[65] Rogers E (1983). Diffusion of innovations.

[66] Green L, Kreuter M (2005). Health program planning: an educational and ecological approach.

[67] West R, Michie S, Rubin GJ, Amlôt R (2020). Applying principles of behaviour change to reduce SARS-CoV-2 transmission. Nat Hum Behav.

[68] Michie S, Van Stralen MM, West R. The behaviour change wheel: a new method for characterising and designing behaviour change interventions. Implement Sci. 2011;6. 10.1186/1748-5908-6-42 PMC3096582 21513547

[69] Michie S, Prestwich A (2010). Are interventions theory-based? Development of a theory coding scheme. Health Psychol.

[70] Michie S, West R, Amlot R, Rubin J. Slowing down the covid-19 outbreak: changing behaviour by understanding it. BMJ Opinion. 2020; https://blogs.bmj.com/bmj/2020/03/11/slowing-down-the-covid-19-outbreak-changing-behaviour-by-understanding-it/. Accessed 8 Aug 2020.

[71] The British Psychological Society (2020). Behavioural science and disease prevention: psychological guidance.

[72] Durham DP, Casman EA (2011). Incorporating individual health-protective decisions into disease transmission models: a mathematical framework. J R Soc Interface.

[73] Funk S, Bansal S, Bauch CT, Eames KT, Edmunds WJ, Galvani AP (2015). Nine challenges in incorporating the dynamics of behaviour in infectious diseases models. Epidemics..

