# Supplementary material for: Examining the application of behaviour change theories in the context of infectious disease outbreaks and emergency response: a review of reviews
Source: BMC Public Health. 2020 Oct 1;20:1483. doi: 10.1186/s12889-020-09519-2 (PMC7528712; doi:10.1186/s12889-020-09519-2)
Supplement: Supplementary file 1 — Additional file 1: Supplementary Information 1. Search terms for the original selection process [file 12889_2020_9519_MOESM1_ESM.docx]

**Supplementary Information 1**

**Embase Search History**:

1. EMBASE; BEHAVIOR/ OR HEALTH BEHAVIOR/; 170249 results.

2. EMBASE; behavio*.ti,ab; 995989 results.

3. EMBASE; COMMUNICABLE DISEASE/; 18203 results.

4. EMBASE; (communic* adj3 disease*).ti,ab; 8964 results.

5. EMBASE; DISEASE TRANSMISSION/; 88854 results.

6. EMBASE; (disease* adj3 transmi*).ti,ab; 32271 results.

7. EMBASE; contagio*.ti,ab; 10073 results.

8. EMBASE; (infecti* adj3 disease*).ti,ab; 117111 results.

9. EMBASE; (protect* adj3 action*).ti,ab; 7813 results.

10. EMBASE; (emergenc* adj3 respon*).ti,ab; 5359 results.

11. EMBASE; (emergenc* adj3 health adj3 prepar*).ti,ab; 242 results.

12. EMBASE; (emergenc* adj3 resilien*).ti,ab; 36 results.

13. EMBASE; (personal adj3 protect* adj3 measure*).ti,ab; 403 results.

14. EMBASE; (adapt* adj3 behavio*).ti,ab; 8359 results.

15. EMBASE; ADAPTIVE BEHAVIOR/; 50451 results.

16. EMBASE; EMERGENCY/; 43582 results.

17. EMBASE; emergenc*.ti,ab; 338859 results.

18. EMBASE; (chemical adj3 terrorism*).ti,ab; 196 results.

19. EMBASE; CHEMICAL WARFARE/; 2175 results.

20. EMBASE; BIOLOGICAL WARFARE/; 8076 results.

21. EMBASE; (bio* adj3 terrorism*).ti,ab; 375 results.

22. EMBASE; (agr* adj3 terrorism*).ti,ab; 14 results.

23. EMBASE; TERRORISM/; 7361 results.

24. EMBASE; (nuclear adj3 terrorism*).ti,ab; 136 results.

25. EMBASE; (disease* adj3 outbreak*).ti,ab; 7273 results.

26. EMBASE; EPIDEMIC/; 82427 results.

27. EMBASE; epidemic*.ti,ab; 83560 results.

28. EMBASE; PANDEMIC/ OR PANDEMIC INFLUENZA/; 11246 results.

29. EMBASE; pandemic*.ti,ab; 22746 results.

30. EMBASE; INFLUENZA/; 50443 results.

31. EMBASE; influenza*.ti,ab; 103325 results.

32. EMBASE; THEORETICAL MODEL/; 72596 results.

33. EMBASE; (theoretic* adj3 model*).ti,ab; 20588 results.

34. EMBASE; HEALTH BELIEF MODEL/; 628 results.

35. EMBASE; (health adj3 belief adj3 model*).ti,ab; 1795 results.

36. EMBASE; HBM.ti,ab; 1093 results.

37. EMBASE; (theor* adj3 planned adj3 behavio*).ti,ab; 2186 results.

38. EMBASE; THEORY OF PLANNED BEHAVIOR/; 809 results.

39. EMBASE; TPB.ti,ab; 1305 results.

40. EMBASE; (social adj3 cog* adj3 theor*).ti,ab; 1671 results.

41. EMBASE; SOCIAL COGNITIVE THEORY/; 827 results.

42. EMBASE; SCT.ti,ab; 12252 results.

43. EMBASE; (extended adj3 parallel adj3 process adj3 model*).ti,ab; 74 results.

44. EMBASE; EPPM.ti,ab; 58 results.

45. EMBASE; (transtheoretical adj3 model*).ti,ab; 1322 results.

46. EMBASE; TTM.ti,ab; 1266 results.

47. EMBASE; (stage* adj3 change*).ti,ab; 7719 results.

48. EMBASE; (theor* adj3 reasoned adj3 action*).ti,ab; 469 results.

49. EMBASE; TRA.ti,ab; 3609 results.

50. EMBASE; (protection adj3 motivation adj3 theor*).ti,ab; 175 results.

51. EMBASE; PMT.ti,ab; 1658 results.

52. EMBASE; REVIEW/; 2058668 results.

54. EMBASE; META ANALYSIS/; 104478 results.

55. EMBASE; (meta adj3 analys*).ti,ab; 110055 results.

56. EMBASE; 1 OR 2; 1065214 results.

57. EMBASE; 3 OR 4 OR 5 OR 6 OR 7 OR 8 OR 9 OR 10 OR 11 OR 12 OR 13 OR 14 OR 15 OR 16 OR 17 OR 18 OR 19 OR 20 OR 21 OR 22 OR 23 OR 24 OR 25 OR 26 OR 27 OR 28 OR 29 OR 30 OR 31; 877177 results.

58. EMBASE; 32 OR 33 OR 34 OR 35 OR 36 OR 37 OR 38 OR 39 OR 40 OR 41 OR 42 OR 43 OR 44 OR 45 OR 46 OR 47 OR 48 OR 49 OR 50 OR 51; 120359 results.

59. EMBASE; 52 OR 53 OR 54 OR 55; 3204723 results.

60. EMBASE; 56 AND 57 AND 58 AND 59; 204 results.

61. EMBASE; 60 [Limit to: (Languages English)]; 196 results.

**PsychInfo Search History**: 1. PsycInfo; BEHAVIOR/ OR BEHAVIORAL SCIENCES/ OR HEALTH BEHAVIOR/; 48682 results.

2. PsycInfo; behavio*.ti,ab; 759565 results.

3. PsycInfo; (communic* adj3 disease*).ti,ab; 982 results.

4. PsycInfo; DISEASE TRANSMISSION/; 1676 results.

5. PsycInfo; (disease* adj3 transmi*).ti,ab; 3964 results.

6. PsycInfo; contagio*.ti,ab; 2407 results.

7. PsycInfo; (infecti* adj3 disease*).ti,ab; 3731 results.

8. PsycInfo; EMERGENCY PREPAREDNESS/; 820 results.

9. PsycInfo; (protect* adj3 action*).ti,ab; 674 results.

10. PsycInfo; (emergenc* adj3 respon*).ti,ab; 1427 results.

11. PsycInfo; (emergenc* adj3 health adj3 prepar*).ti,ab; 53 results.

12. PsycInfo; (emergenc* adj3 resilien*).ti,ab; 38 results.

13. PsycInfo; (personal adj3 protect* adj3 measure*).ti,ab; 16 results.

14. PsycInfo; ADAPTIVE BEHAVIOR/; 2481 results.

15. PsycInfo; (adapt* adj3 behavio*).ti,ab; 10497 results.

16. PsycInfo; emergenc*.ti,ab; 45594 results.

17. PsycInfo; (chemical adj3 terrorism*).ti,ab; 26 results.

18. PsycInfo; (bio* adj3 terrorism*).ti,ab; 42 results.

19. PsycInfo; (agr* adj3 terrorism*).ti,ab; 8 results.

20. PsycInfo; (nuclear adj3 terrorism*).ti,ab; 36 results.

21. PsycInfo; (disease* adj3 outbreak*).ti,ab; 238 results.

22. PsycInfo; EPIDEMICS/; 2452 results.

23. PsycInfo; epidemic*.ti,ab; 9541 results.

24. PsycInfo; PANDEMICS/; 418 results.

25. PsycInfo; pandemic*.ti,ab; 1484 results.

26. PsycInfo; INFLUENZA/ OR SWINE INFLUENZA/; 1194 results.

27. PsycInfo; influenza*.ti,ab; 2063 results.

28. PsycInfo; MODELS/; 57436 results.

29. PsycInfo; (theoretic* adj3 model*).ti,ab; 18912 results.

30. PsycInfo; (health adj3 belief adj3 model*).ti,ab; 1504 results.

31. PsycInfo; HBM.ti,ab; 378 results.

32. PsycInfo; (theor* adj3 planned adj3 behavio*).ti,ab; 3385 results.

33. PsycInfo; TPB.ti,ab; 1470 results.

34. PsycInfo; (social adj3 cog* adj3 theor*).ti,ab; 4249 results.

35. PsycInfo; SCT.ti,ab; 854 results.

36. PsycInfo; (extended adj3 parallel adj3 process adj3 model*).ti,ab; 104 results.

37. PsycInfo; EPPM.ti,ab; 56 results.

38. PsycInfo; (transtheoretical adj3 model*).ti,ab; 1548 results.

39. PsycInfo; TTM.ti,ab; 681 results.

40. PsycInfo; STAGES OF CHANGE/; 813 results.

41. PsycInfo; (stage* adj3 change*).ti,ab; 4049 results.

42. PsycInfo; (theor* adj3 reasoned adj3 action*).ti,ab; 1215 results.

43. PsycInfo; TRA.ti,ab; 1075 results.

44. PsycInfo; (protection adj3 motivation adj3 theor*).ti,ab; 287 results.

45. PsycInfo; PMT.ti,ab; 362 results.

46. PsycInfo; review*.ti,ab; 445630 results.

47. PsycInfo; META ANALYSIS/; 15738 results.

48. PsycInfo; (meta adj3 analys*).ti,ab; 21074 results.

49. PsycInfo; 1 OR 2; 768634 results.

50. PsycInfo; 3 OR 4 OR 5 OR 6 OR 7 OR 8 OR 9 OR 10 OR 11 OR 12 OR 13 OR 14 OR 15 OR 16 OR 17 OR 18 OR 19 OR 20 OR 21 OR 22 OR 23 OR 24 OR 25 OR 26 OR 27; 81010 results.

51. PsycInfo; 28 OR 29 OR 30 OR 31 OR 32 OR 33 OR 34 OR 35 OR 36 OR 37 OR 38 OR 39 OR 40 OR 41 OR 42 OR 43 OR 44 OR 45; 88657 results.

52. PsycInfo; 46 OR 47 OR 48; 458955 results.

53. PsycInfo; 49 AND 50 AND 51 AND 52; 163 results.

54. PsycInfo; 53 [Limit to: (Language English)]; 156 results.

**Medline Search History**:

1. Medline; BEHAVIOR/ OR BEHAVIOR AND BEHAVIOR MECHANISMS/ OR BEHAVIORAL RESEARCH/ OR BEHAVIORAL SCIENCES/ OR HEALTH BEHAVIOR/; 68250 results.

2. Medline; behavio*.ti,ab; 867526 results.

3. Medline; COMMUNICABLE DISEASE CONTROL/ OR COMMUNICABLE DISEASES/; 40785 results.

4. Medline; (communic* adj3 disease*).ti,ab; 8205 results.

5. Medline; DISEASE TRANSMISSION, INFECTIOUS/; 6598 results.

6. Medline; (disease* adj3 transmi*).ti,ab; 28612 results.

7. Medline; contagio*.ti,ab; 9182 results.

8. Medline; (infecti* adj3 disease*).ti,ab; 99179 results.

9. Medline; (protect* adj3 action*).ti,ab; 7808 results.

10. Medline; (emergenc* adj3 respon*).ti,ab; 5207 results.

11. Medline; (emergenc* adj3 health adj3 prepar*).ti,ab; 306 results.

12. Medline; (emergenc* adj3 resilien*).ti,ab; 37 results.

13. Medline; (personal adj3 protect* adj3 measure*).ti,ab; 356 results.

14. Medline; (adapt* adj3 behavio*).ti,ab; 8054 results.

15. Medline; emergenc*.ti,ab; 255961 results.

16. Medline; (chemical adj3 terrorism*).ti,ab; 177 results.

17. Medline; CHEMICAL TERRORISM/; 139 results.

18. Medline; (bio* adj3 terrorism*).ti,ab; 234 results.

19. Medline; BIOTERRORISM/; 4425 results.

20. Medline; (agr* adj3 terrorism*).ti,ab; 13 results.

21. Medline; (nuclear adj3 terrorism*).ti,ab; 122 results.

22. Medline; (disease* adj3 outbreak*).ti,ab; 8197 results.

23. Medline; DISEASE OUTBREAKS/; 67083 results.

24. Medline; EPIDEMICS/; 6464 results.

25. Medline; epidemic*.ti,ab; 74793 results.

26. Medline; pandemic*.ti,ab; 18557 results.

27. Medline; INFLUENZA PANDEMIC, 1918-1919/ OR PANDEMICS/; 3788 results.

28. Medline; INFLUENZA, HUMAN/; 40196 results.

29. Medline; influenza*.ti,ab; 95743 results.

30. Medline; MODELS, THEORETICAL/; 117975 results.

31. Medline; (theoretic* adj3 model*).ti,ab; 22579 results.

32. Medline; (health adj3 belief adj3 model*).ti,ab; 1603 results.

33. Medline; HBM.ti,ab; 889 results.

34. Medline; (theor* adj3 planned adj3 behavio*).ti,ab; 1986 results.

35. Medline; TPB.ti,ab; 1148 results.

36. Medline; (social adj3 cog* adj3 theor*).ti,ab; 1526 results.

37. Medline; SCT.ti,ab; 6575 results.

38. Medline; (extended adj3 parallel adj3 process adj3 model*).ti,ab; 82 results.

39. Medline; EPPM.ti,ab; 56 results.

40. Medline; (transtheoretical adj3 model*).ti,ab; 1179 results.

41. Medline; TTM.ti,ab; 916 results.

42. Medline; (stage* adj3 change*).ti,ab; 9076 results.

43. Medline; (theor* adj3 reasoned adj3 action*).ti,ab; 427 results.

44. Medline; TRA.ti,ab; 7454 results.

45. Medline; (protection adj3 motivation adj3 theor*).ti,ab; 171 results.

46. Medline; PMT.ti,ab; 1346 results.

47. Medline; REVIEW/; 0 results.

48. Medline; review*.ti,ab; 1438537 results.

49. Medline; META-ANALYSIS/; 0 results.

50. Medline; (meta adj3 analys*).ti,ab; 83660 results.

51. Medline; 1 OR 2; 901203 results.

52. Medline; 3 OR 4 OR 5 OR 6 OR 7 OR 8 OR 9 OR 10 OR 11 OR 12 OR 13 OR 14 OR 15 OR 16 OR 17 OR 18 OR 19 OR 20 OR 21 OR 22 OR 23 OR 24 OR 25 OR 26 OR 27 OR 28 OR 29; 637298 results.

53. Medline; 30 OR 31 OR 32 OR 33 OR 34 OR 35 OR 36 OR 37 OR 38 OR 39 OR 40 OR 41 OR 42 OR 43 OR 44 OR 45 OR 46; 168267 results.

54. Medline; 47 OR 48 OR 49 OR 50; 1479657 results.

55. Medline; 51 AND 52 AND 53 AND 54; 116 results.

56. Medline; 55 [Limit to: (Language English)]; 112 results.
