# Supplementary material for: Examining the application of behaviour change theories in the context of infectious disease outbreaks and emergency response: a review of reviews
Source: BMC Public Health. 2020 Oct 1;20:1483. doi: 10.1186/s12889-020-09519-2 (PMC7528712; doi:10.1186/s12889-020-09519-2)
Supplement: Supplementary file 2 — Additional file 2: Supplementary Information 2. Summary of key theory-related conclusions [file 12889_2020_9519_MOESM2_ESM.docx]

**Supplementary Information 2**

**Summaries of Key Theory-Related Conclusions**

*Angus, Cairns, Purves, Bryce, MacDonald, & Gordon (2013)*

All studies explicitly used interventions based on or informed by theory or models. Individual health-related behavioural models were most commonly used, such as Health Belief Model, Theory of Reasoned Action, Theory of Planned Behaviour. Social Cognitive Theory (or Social Learning Theory, its predecessor) also frequently cited.

Similarities were observed in the theories used for both successful and unsuccessful interventions, the review authors do not note any substantive differences in the theories used for effective and ineffective interventions. A substantial difference is that those studies citing the use of a theory in intervention design were more likely to be effective than those who used a theory for intervention evaluation.

The absence of comparative evidence means that the review authors cannot draw conclusions concerning the impact of theory on intervention effectiveness. However, they do conclude that there is a large evidence base of successful interventions based on theory.

There is little to no systematic critique within the literature concerning the applicability of theories within the evidence base. The review authors conclude that it is important to ensure that suitable theories are used for intervention design (based on the specific problem and associated factors).

The review authors recommend researchers look beyond individual-level theories to look at theories which account for community-level behaviour.

The review authors further recommend additional research to advance understanding of the relationships between key variables and health behaviours. This may, according to the review authors, provide insight into why multiple theoretical approaches may be effective or ineffective.

*Bish & Michie (2010)*

Review findings can be broadly explained by behaviour change theories.

Support for both Health Belief Model and Protection Motivation Theory in terms of the roles of attitudes, risk perception and efficacy for behaviour change.

Criticism of Health Belief Model, Protection Motivation Theory, and Theory of Planned Behaviour as they do not adequately consider emotional factors. Review authors suggest support for parallel processing models such as the Common Sense Model of Illness for representing emotional factors.

Review authors also suggest support for the two-system model of behaviour and PRIME theory of motivation in understanding the role of anxiety.

Further research should be theory-driven and is required to study the relationships between demographics, attitudes, and behaviour. Results from this can subsequently be used to develop interventions.

Review authors discuss limitation of social cognitive models for intervention design (citing Sniehotta, 2009).

*Bish & Michie (2011)*

Only one study cited by the review authors was based on an explicit theoretical framework.

Echoes Bish and Michie (2010) but essentially supports the relevance of theories including the Health Belief Model, Theory of Planned Behaviour, Protection Motivation Theory, and the Common Sense Model of Illness.

*Bish, Michie & Yardley (2011)*

Most studies did not explicitly apply theory. However, review findings (concerning perceived susceptibility, threat, and vaccine safety concerns) can be understood in terms of theories such as the Health Belief Model, Theory of Planned Behaviour, Protection Motivation Theory and the Common Sense Model of Illness

There is a lack of theoretical evidence in the development and evaluation of interventions. Future interventions need to be developed and evaluated with reference to theory.

*Bish, Yardley, Nicoll & Michie (2011)*

Findings consistent with Protection Motivation Theory in terms of the relationships between threat and perceptions of vaccine and behavioural outcomes (intentions and uptake)

There is a lack of theory within research.

The review authors indicate a need for the use of theory in future research and intervention design

*Bults, Beaujean, Richardus & Voeten (2015)*

Review presents the primary constructs of Protection Motivation Theory but also mentions other constructs from different theories that may influence behaviour: barriers, benefits, social norms, trust.

Low number of papers describe behavioural theories in the study rationale or questionnaire development.

Review doesn’t really speak to the efficacy of particular theories but does advocate the use of theories in design.

*Corace, Srigley, Hargadon, Yu, MacDonald, Fabrigar, & Garber (2016)*

The Health Belief Model was the most commonly applied theory and was strongly predictive of vaccinated behaviour among healthcare workers (all components).

Theory of Planned Behaviour was successful in one of the two cited papers, the review authors cite it as well-suited for vaccination behaviour.

The review authors note Risk Perception Attitude Framework and the Triandis Model of Interpersonal Behaviour as having promise, but with further research needed.

However, the data for behaviour framework utility is not translated into the use of these frameworks for intervention design. No interventions based on psychological theories of behaviour change were identified within the review.

*Ejeta, Ardalan, & Paton (2015)*

Health Belief Model, Extended Parallel Processing Model, Theory of Planned Behaviour and Social Cognitive Model most commonly used. Overall, theories and models applied in this context provide strong evidence and can guide individuals aiming to develop interventions.

Support for Health Belief Model for predicting behaviour in both a disease outbreak context and climate change context. However, less support was found for the Health Belief Model in the context of preparedness for heat wave hazards. There may therefore be variable effects depending on hazard. Support for the role of Theory of Planned Behaviour in the context of disease outbreak preparedness. Social Cognitive Model supported by research using path analysis or structural equation modelling to examine relationship between variables.

Support for Extended Parallel Process Model in the context of disaster and emergency preparedness. Review authors remark that the role of fear in the cited findings further supports the need to extend the application of the Extended Parallel Process Model beyond the inclusion of self-efficacy and threat.

For multiple theories the review authors note that evidence for the association between theoretical constructs and preparedness is inconsistent. Review authors conclude that structural equation modelling and path analyses should be used (rather than traditional logistic regression analyses frequently depicted) in order to represent the interconnected and interdependent relationship between constructs.

*Leppin & Aro (2009)*

Slightly over 1/3 of studies refer to a theoretical model, the theoretical model most cited was the Health Belief Model.

The review authors conclude that there is not enough use of theory to define or operationalise risk, to understand the theoretically-supported relationship between likelihood and severity, or to fully explore the relationship between risk perception and behaviour.

More systematic application of multivariable models rather than just correlations between single concepts was therefore recommended by the review authors. Indeed, the review authors recommend that researchers conduct more systematic and theory-driven work to examine pandemic influenza risk perception, to further advance concept development and measurement.

*Omori, Kuligowski, Butler, & Gwynne (2017)*

The Protective Action Decision Model is used as framework for the literature review.

Human behaviour theory (unspecified) was used to resolve any contradictory findings that arose during the review process.

The Protective Action Decision Model was appraised by the authors as being both detailed enough to represent the crucial components of human decision-making and also simple enough to be of use to practitioners.

*Prematunge, Corace, McCarthy, Nair, Pugsley, & Garber (2012)*

Support for the Health Belief Model in terms of cues to action, perceived barriers, perceived benefits, perceived severity, and perceived susceptibility.

Support for the Health Belief Model as a theory for understanding healthcare worker pandemic vaccination

*Schmid, Rauber, Betsch, Lidold, & Denker (2017)*

On both a micro level (Theory of Planned Behaviour) and macro level (4Cs) the theories identified seem to be of relevance for categorising the findings within cited literature. On a micro-level, overall, attitudes, utility of vaccine, cues to action, and previous behaviour are key. On a macro level, confidence (safety and effectiveness of the vaccine, trust) and complacency (risk, worry, severity) are key.

*Westcott, Ronan, Bambrick, & Taylor (2017)*

The authors review previous uses of Protection Motivation Theory and suggest an application and extension to help emergency responders understand the behaviour of animal owners in relation to bushfires.

Two limitations are raised regarding the application of Protection Motivation Theory in this context. Specifically, a lack of familiarity with the theory (and potential scepticism concerning the use of academic theory in practice), and the potential role of emotional attachment (to the animals) in overriding adaptive responding.

Overall, the authors conclude that Protection Motivation Theory has been established as a robust, successful and adaptable theory, that has been applied across a wide range of contexts throughout its history. The authors further acknowledge the simplicity and flexibility of Protection Motivation Theory as key strengths for its practical applicability generally, and specifically for its application and extension in the context of bushfire preparedness.
